# Allogeneic hematopoietic cell transplantation, the microbiome, and graft-versus-host disease

**DOI:** 10.1080/19490976.2023.2178805

**Published:** 2023-02-15

**Authors:** Yannouck F. van Lier, Jaël Vos, Bianca Blom, Mette D. Hazenberg

**Affiliations:** a Department of Hematology, Amsterdam UMC location AMC, Amsterdam, The Netherlands; b Department of Experimental Immunology, Amsterdam Institute for Infection & Immunity Institute, Cancer Center Amsterdam, Amsterdam UMC location AMC, Amsterdam, The Netherlands; c Department of Hematopoiesis, Sanquin Research, Amsterdam, The Netherlands

**Keywords:** Allogeneic HCT, GvHD, gut microbiome, gut microbiota, FMT, mucosal immune system

## Abstract

Many patients with hematological malignancies, such as acute myeloid leukemia, receive an allogeneic hematopoietic cell transplantation (HCT) to cure their underlying condition. Allogeneic HCT recipients are exposed to various elements during the pre-, peri- and post-transplant period that can disrupt intestinal microbiota, including chemo- and radiotherapy, antibiotics, and dietary changes. The dysbiotic post-HCT microbiome is characterized by low fecal microbial diversity, loss of anaerobic commensals, and intestinal domination, particularly by *Enterococcus* species, and is associated with poor transplant outcomes. Graft-versus-host disease (GvHD) is a frequent complication of allogeneic HCT caused by immunologic disparity between donor and host cells and results in tissue damage and inflammation. Microbiota injury is particularly pronounced in allogeneic HCT recipients who go on to develop GvHD. At present, manipulation of the microbiome for example, via dietary interventions, antibiotic stewardship, prebiotics, probiotics, or fecal microbiota transplantation, is widely being explored to prevent or treat gastrointestinal GvHD. This review discusses current insights into the role of the microbiome in GvHD pathogenesis and summarizes interventions to prevent and treat microbiota injury.

## Introduction

Allogeneic hematopoietic cell transplantation (HCT) is used as consolidation therapy for many hematologic malignancies with the goal to induce a graft-versus-leukemia or graft-versus-lymphoma (GvL) response via alloreactive donor lymphocytes that eliminate residual tumor cells of the patient. These alloreactive lymphocytes, however, often also target the recipients’ tissues, inducing an inflammatory syndrome known as graft-versus-host disease (GvHD).^1^ GvHD is one of the major contributors to the high costs, the high morbidity, and the 10–30% transplantation-related mortality of allogeneic HCT, in addition to relapse of primary disease, organ failure, and infections.^2–5^ Treatment for GvHD consists of corticosteroids (prednisolone) and other immunosuppressants, such as the macrolide sirolimus, calcineurin inhibitors, and Janus-kinase-2 inhibitors such as ruxolitinib. Patients with steroid-refractory acute GvHD have a dismal prognosis.^6^ In search of new therapeutic strategies to improve the outcome for allogeneic HCT recipients, the intestinal microbiome has rapidly gained attention over the past decade. This review focuses specifically on the role of the intestinal microbiome in the development of GvHD and speculates on pre-transplant strategies to preserve a healthy microbiome or post-transplant therapies that target the gut flora to prevent or treat GvHD.

### The pathogenesis of graft-versus-host disease

GvHD is classified into acute GvHD that develops early (<100 days) after allogeneic HCT, and late-onset acute or chronic GvHD that are considered late (>100 days) transplant-related complications. Acute GvHD primarily involves the skin, liver, and gastrointestinal tract, where symptoms typically include skin rash, jaundice, nausea, cramping, and diarrhea.^7^ Chronic GvHD is predominantly a sclerosing disease that can virtually affect any organ. Allogeneic HCT recipients routinely receive prophylactic immunosuppression, comprising (a combination of) anti-thymocyte globulin (ATG), post-transplantation cyclophosphamide, calcineurin inhibitors, mycophenolic acid, or methotrexate to prevent GvHD but despite these efforts, acute GvHD develops in 30–50% of the allogeneic HCT recipients.

**Figure 1.**
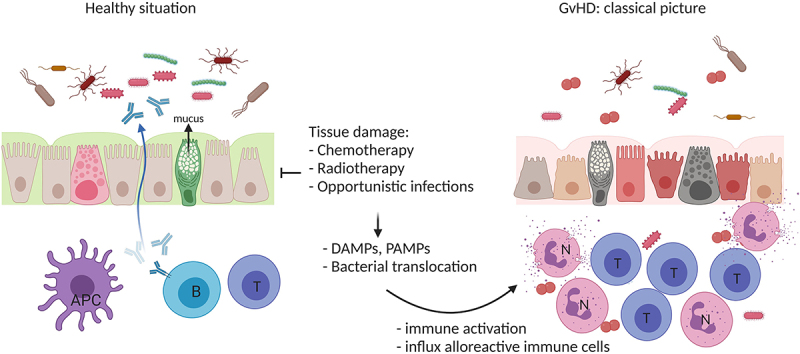
**The classical picture of GvHD pathophysiology**. Chemotherapy and/or radiotherapy (host conditioning) results in tissue damage, translocation of bacteria, and the release of damage-associated molecular patterns (DAMP) and pathogen-associated molecular patterns (PAMP), which activate host antigen-presenting cells (APC). APCs stimulate alloreactive donor lymphocytes (e.g. T cells (t)), which in turn produce inflammatory cytokines and recruit additional effector cells (e.g. neutrophils (n)) resulting in enhanced tissue damage and inflammation. *Created with BioRender.com.*

In the classical view of acute GvHD pathophysiology, alloreactivity, and tissue damage are considered key driving factors (Figure 1).^7–9^ Chemo(radio)therapy preceding allogeneic HCT (referred to as ‘conditioning therapy’ as it conditions the recipient to receive the allograft) induces tissue damage and inflammation of the intestinal mucosa (mucositis), thereby weakening the intestinal mucosal barrier and increasing the risk of bacterial translocation. Together, this leads to the release of damage-associated molecular patterns (DAMPs) and pathogen-associated molecular patterns (PAMPs) which activate hematopoietic and non-hematopoietic antigen-presenting cells (APCs). These APCs produce inflammatory cytokines and promote the priming and differentiation of donor immune cells. Activated neutrophils and alloreactive lymphocytes, including effector cells that are recruited into the tissues, also release inflammatory cytokines that enhance tissue damage and inflammation. It is assumed that chronic GvHD and the curative GvL response develop along the same pathways.^10^

### Evidence for a role for microbiota in GvHD pathophysiology and other transplantation-related complications

In steady-state conditions, a diverse collection of anaerobic commensal bacteria prevents the outgrowth of potential pathogens, produces essential nutrients, and orchestrates intestinal homeostasis through reciprocal interactions with the mucosal immune system.^11^ Gut microbiota contributes to a tolerant intestinal mucosal immune system for example, via the degradation of dietary polysaccharides into short-chain fatty acids (SCFA), such as butyrate, propionate, and acetate, which have an immunomodulatory function.^12^ SCFA stimulates the production of anti-inflammatory interleukin (IL)-10 by regulatory T cells (Treg, via inhibition of histone deacetylation) and IL-22 by both CD4^+^ T cells and innate lymphoid cells (ILC), which enhance epithelial integrity.^13–15^ SCFA also promote immunoglobulin A (IgA) secretion by B cells that provides a first line of defense against infiltrating pathogens.^16^ Furthermore, SCFA and other bacterial metabolites such as polyamines enhance gut barrier function by inducing Goblet cells to produce mucus and by stimulating the production of endothelial cell tight junction proteins.^17,18^

Advances in sequencing technology have allowed rapid and large-scale exploration of the compositional and to some extent functional configuration of the microbial communities in the human gut. This has initiated an impressive number of studies focusing on the relationship between microbiota and human health and led to the awareness that damage to the enteric bacterial population is associated with a variety of immune-mediated diseases, including GvHD.^19^ In 2012, it was reported that microbial diversity is reduced early after allogeneic HCT,^20^ and that this loss of diversity preceded the development of acute GvHD.^21^ A larger, prospective study later demonstrated that patients who maintained microbiota diversity had a significantly reduced probability of death due to GvHD or opportunistic infections, which translated into a better overall survival than patients who experienced loss of microbiome diversity.^22^ This association between low microbial diversity and survival disadvantage, more specifically higher transplantation-related and GvHD-related mortality, was recently confirmed in an international, multicenter study that included over 8000 samples from 1362 unique patients.^23^ Interestingly, this association was less apparent in patients receiving a lymphocyte-depleted allograft. The presence of alloreactive (donor-derived) adaptive immune cells thus appears to be a major contributor to the mortality associated with microbiota disruption. Indeed, in a murine allogeneic HCT model, it was demonstrated that radiotherapy-induced bacterial translocation leads to the recruitment of neutrophils and then, via the activation of alloreactive T cells, to acute GvHD.^24^

Loss of microbiome diversity may play a role in other aspects complicating transplantation outcomes that were noted in the past but less well understood. For example, obesity is associated with adverse outcome after allogeneic HCT. With the help of a murine HCT model, it was demonstrated that obesity, with its associated lack of microbiome diversity, leads to a pro-inflammatory intestinal milieu and an increased risk for GvHD.^25^ Moreover, allogeneic HCT recipients who survive the first 1–2 years after transplantation maintain a life-long increased risk for cardiovascular events and secondary malignancies. Loss of microbiome diversity is associated with a risk of cardiovascular events, malignancies, and poor health in general.^26^ In a cross-sectional study, it was demonstrated that the microbiome of allogeneic HCT survivors who developed secondary malignancies was more perturbed than long-term survivors without secondary malignancies.^27^ It is safe to assume that long-term complications in allogeneic HCT survivors are related to microbiome damage encountered early after transplantation, but evidence is lacking thus far.

### Factors involved in microbiome disruption

**Figure 2.**
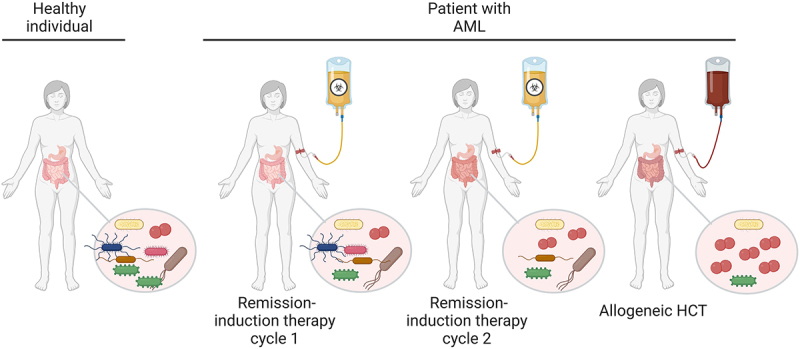
**Peri-transplant microbiome injury**. A typical schedule for the treatment of acute myeloid leukemia (AML) is depicted, with two AML remission-induction chemotherapy cycles followed by allogeneic HCT. Repeated cycles of remission-induction chemotherapy and subsequent allogeneic HCT are associated with alterations in diet and necessitate the use of antibiotics, anti-emetics, and other drugs that lead to loss of microbiome diversity. In addition, chemotherapy and radiotherapy directly damage the microbiome. *Created with BioRender.com.*

Intestinal microbial diversity starts to decrease prior to transplantation, during the phase of remission-induced chemotherapy, and continues to decline in the first days after transplantation (Figure 2).^20,28,29^ The post-HCT intestinal microbiome commonly features low diversity, decreased abundance of commensals, and a high frequency of *Enterococcus, Streptococcus*, or Proteobacteria colonization compared to the microbiome of healthy individuals.^23^ A number of factors have been linked to early microbiota injury during allogeneic HCT. Allogeneic HCT recipients typically receive multiple rounds of chemotherapy, sometimes combined with total body irradiation (TBI), to reduce tumor burden and eliminate the recipients’ adaptive immune system to prevent graft rejection. The toxicity of both therapies has been implicated in the loss of microbial diversity and modulation of microbiome composition.^30–36^ This toxicity may act directly on gut microbes, and indirectly for example, through the death of Paneth cells. Paneth cells are secretory cells located at the intestinal crypt base where they produce antimicrobial factors to preserve host-microbial homeostasis.^37^ Patients with acute GvHD have reduced numbers of intestinal Paneth cells, while rescue of Paneth cells via treatment with glucagon-like-peptide-2 restored microbiome damage in a GvHD mouse model.^38^

Most studied to date, however, is the microbiome-damaging effect of antibiotics, generally considered the culprit (but certainly not the only cause) of microbiome damage in allogeneic HCT recipients. Exposure to antibiotics is high and prolonged in allogeneic HCT recipients. Chemotherapy-induced mucositis of the oral cavity and gastrointestinal tract increases the risk of bacterial translocation,^39^ while remission-induction and conditioning chemotherapy regimens frequently lead to neutropenia. Prophylactic antibiotics are therefore routinely prescribed to prevent systemic infections, and patients receive empiric broad-spectrum antibiotics during episodes of neutropenic fever. In a retrospective study, the early administration of broad-spectrum antibiotics (between day −7 and day 0 before allogeneic HCT) led to a reduction in the abundance of *Clostridiales* and was associated with higher transplant-related mortality.^40^ Furthermore, the use of certain antibiotics was associated with a higher incidence of intestinal colonization and subsequent bloodstream infections by the respective dominating species.^20,41^ Antibiotics are, however, not the only drugs that can negatively impact microbiome diversity. Almost any drug can disturb the microbial community, as was demonstrated in two large observational studies in the healthy population of The Netherlands and Belgium.^42,43^ Commonly used drugs like anti-emetics, proton pump inhibitors, antidepressants, and opiates were demonstrated to affect the composition of the microbiome at a population level, and are therefore likely to impact the microbiome in allogeneic HCT recipients.

Another important factor that disturbs intestinal homeostasis may be the prolonged immunodeficiency that is characteristic of allogeneic HCT. The microbiome is shaped by microbes encountered from birth onwards and the interaction of these microbes with the developing immune system of the infant.^44,45^ After allogeneic HCT, this reciprocal interaction between the immune system and the microbiome comes under significant pressure as reconstitution of the donors’ adaptive immune system after transplantation typically takes months to years. This may significantly delay the recovery of the microbiome. In solid organ transplant recipients, the use of immunosuppressive drugs was the single most important determinant of microbiome damage, that lasted for years after transplantation.^46^ In allogeneic HCT, the microbiome was demonstrated to affect the recovery of innate and adaptive immunity in general and T cell recovery specifically, reinforcing the reciprocal nature of the interaction between the immune system and the microbiome.^47,48^

Finally, diet has a major influence on the constituents of the intestinal microbiome.^49,50^ The oral intake of allogeneic HCT recipients is frequently diminished due to the loss of appetite, pain, and nausea that is induced by mucositis, and this often requires parenteral feeding to ensure sufficient caloric intake.^51^ In two small cohorts, parenteral feeding was inferior to enteral nutrition in preserving microbiota diversity and composition.^52,53^ Moreover, two retrospective studies showed that parenteral nutrition was associated with a higher incidence of gastro-intestinal GvHD and lower survival rates compared to patients receiving adequate enteral nutrition.^54,55^

**Figure 3.**
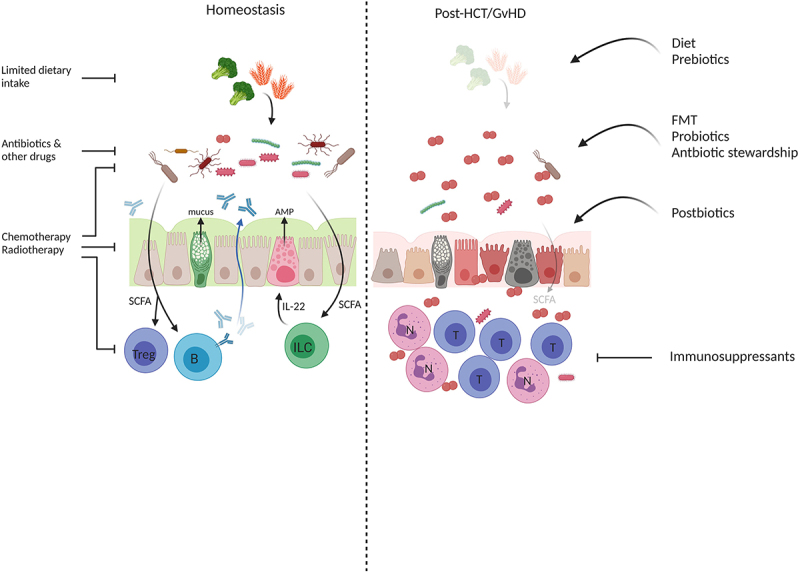
**Disruption and restoration of intestinal homeostasis in GvHD**. In the healthy situation (left panel), commensals metabolize dietary fibers to short-chain fatty acids (SCFA), which are important for immunologic tolerance via induction of regulatory T cells (Treg) and the production of secretory IgA by B cells (b). SFCA enhance IL-22 production via innate lymphoid cells (ILC) thereby supporting epithelial integrity and promoting anti-microbial peptide (AMP) production that are important in shaping the microbial community and the prevention of pathogen outgrowth. Goblet cells produce mucus to hamper bacterial translocation. All of these processes are impacted by factors that are inherent in cancer treatment, such as the use of antibiotics, changes in diet, etcetera. This results in dysbiosis, low levels of SCFA, hampered mucus production, damage to epithelial cells, activation and the influx of (alloreactive) T cells (t) and neutrophils (n), and inflammation (right panel). Where classic GvHD treatment predominantly focuses on tempering immune activation via immunosuppressants, novel approaches include therapies that target the microbiota to prevent or treat dysbiosis. *Created with BioRender.com.*

Collectively, these factors can drastically alter the gut microbiota community and disrupt intestinal homeostasis. The insights obtained from the different studies mentioned above led to a revised model of GvHD pathophysiology, where the importance of the gut microbiota as a mediator of immunologic tolerance and keeper of gut homeostasis was incorporated (Figure 3).^56^

### The ‘good’ and ‘bad’ guys associated with post-transplant complications

With the recognition of the intestinal microbiome as an important factor in GvHD pathogenesis, the question was raised whether gut homeostasis is regulated by the community as a whole, or by certain specific species. A number of studies demonstrated that certain species are in particular associated with worse outcome. The facultative anaerobe *Enterococcus* genus, which in the healthy microbiome represents only a very small minority (<0,1%),^57^ might be the best-studied pathobiont in relation to acute GvHD. Enterococci in particular, E. *faecium* and E. *faecalis*, are a prevalent cause of infection^58^ and, even more importantly in the context of GvHD, they have emerged as potent stimulators of intestinal inflammation.^59^ For example, E. *faecalis* compromises gut barrier integrity,^60^ induces the production of inflammatory cytokines,^61^ and causes colitis in IL-10 deficient mice.^62^ E. *faecalis* also induced donor T cell activation in a mouse GvHD model, suggesting a direct causal relationship.^63^ The frequency of intestinal *Enterococcus* domination is high in allogeneic HCT recipients and peaks at 1 week after HCT.^23^ In particular, there is a high abundance of *E. faecium* observed, which correlates with more severe acute GvHD and reduced survival.^63^ Other commonly dominating genera in allogeneic HCT recipients are *Lactobacillus*^28^ and *Streptococcus. Streptococcus* abundance is associated with induction of pro-inflammatory cytokines^64^ and has been shown to impair the growth of commensal bacteria in the oral microbiota.^65^ *Streptococcus* infections are more abundant in allogeneic HCT recipients with GvHD and are associated with higher mortality.^66–68^

Other species have been attributed to protective and/or anti-inflammatory capacities. For example, *Barnesiella*^69^ and *Lactobacillus Johnsonii*^21^ suppressed the outgrowth of *Enterococcus* species and thereby protect microbial homeostasis. A direct association with protection against lethal GvHD in allogeneic HCT recipients has been observed with the presence of *Blautia*, a genus from the Clostridia class.^70^ Preservation of *Blautia* was associated with reconstitution of gut epithelium protective mucosal-associated invariant T (MAIT) cells, which correlated with less acute GvHD and improved survival.^71,72^ An earlier study demonstrated that a mixture of 17 rationally selected Clostridia strains induced accumulation of Tregs^73^ and mitigated GvHD in mice,^74^ suggesting that this class of bacteria are important mediators in preserving intestinal homeostasis. One of the underlying mechanisms could be SCFA production; several Clostridia species, including *Blautia* spp., are well-known butyrate producers. A recent study reported that the fecal SCFA concentration is reduced in acute GvHD patients and correlates to the severity of disease.^75^ The concentration of circulating SCFAs also decreased, specifically in patients that go on to develop chronic GvHD.^76^ Loss of SCFA-producing bacteria and subsequent reduction of their beneficial metabolites thus appears to be important to limit conditioning or GvHD-mediated damage.^74^

### Targeting the microbiome to prevent or treat acute GvHD

Together, these data suggest that maintenance of microbiome diversity in general, and specifically preservation or return of obligate anaerobe commensals, such as *Blautia*, could be of importance to prevent or treat acute GvHD. Given the many factors that affect the microbiome, there are a number of ways to prevent or treat microbiome damage during allogeneic HCT (Figure 3). Most of the studies referenced here are prospective trials or mechanistic (e.g. mouse model) studies, to avoid confounding factors that so often hamper proper interpretation of allogeneic HCT studies.

The most obvious first step is to limit damage to the intestinal microbiota. Antibiotic stewardship can play an important role in sparing the obligate anaerobe commensal community. In a retrospective study that included 161 allogeneic HCT recipients, it was demonstrated that the use of rifaximin preserved microbiota diversity, even when followed by systemic antibiotics.^77^ This was in contrast to other frequently used regimens, such as piperacillin/tazobactam or ciproxin/metronidazole, which were associated with significant microbiota disruption and damage to the intestinal epithelium.^78^ Patients who received rifaximin had a lower incidence of acute and chronic GvHD and better overall survival.^79^ Other studies investigating strategies to optimize antibiotic regimens to preserve intestinal microbiota composition are ongoing (for example, NCT03727113). Alternative strategies to prevent dysbiosis include compounds that neutralize antibiotic residues once they enter the colon^80,81^ and bacteria-specific antibody-antibiotic conjugates.^82^ These compounds have, however, not been tested in the context of allogeneic HCT. Novel approaches, such as CRISPR-Cas9 phagemids targeting pathogens by producing sequence-specific antimicrobials, are being developed.^83^

In healthy individuals, the effect of dietary intake on the microbiome is high and likely depends on the individual microbiome.^50,84^ Data obtained from mice and men demonstrate that *Enterococcus* domination is fed by dietary lactose, suggesting that lactose intake should be limited early after allogeneic HCT to prevent *Enterococcus* domination and acute GvHD.^63^ Thus far, reports on specific dietary interventions in allogeneic HCT are lacking but several studies are underway (for example, NCT0559009).

When microbiome damage cannot be prevented, ‘health-promoting’ species can be selectively or unselectively reintroduced. Probiotics are viable microorganisms that can be directly introduced in an attempt to restore bacterial diversity.^85^ Commercially available probiotics typically contain *Lactobacillus* and *Bifidobacterium* strains. In allogeneic HCT mouse models, administration of *Lactobacillus* rhamnosus GG resulted in less GvHD and increased survival.^86^ However, these findings were not reproducible in a study including allogeneic HCT recipients.^87^ In addition, the use of probiotics does raise concern in allogeneic HCT because of the risk of infectious complications.^88,89,90,91,92^ More importantly perhaps is the observation that probiotic therapy delayed rather than enhanced recovery of microbiome damage in healthy human volunteers.^93^ This raises serious questions regarding the efficacy of such therapy in humans.^93,94^ A rationally selected consortium of bacterial strains for example, containing Clostridia species, could offer an alternative for the commercial probiotics that are currently available. While further research might be needed to determine the optimal composition, the potential of bacterial consortia has been reported in a number of pre-clinical studies.^73,74,95,96^

Unselective introduction of bacterial species can be achieved by fecal microbiota transplantation (FMT). Donor FMT was first used in 1958 to treat colitis^97^ and is now applied to treat recurrent *Clostridioides difficile* infection.^98^ FMT resulted in restoration of microbiota diversity in patients who had received an allogeneic HCT.^99,100^ A few case reports suggested efficacy of FMT in steroid-refractory GvHD.^101–103^ We performed a prospective, single-arm study including 15 steroid-dependent or steroid-refractory GvHD demonstrating complete remission of GvHD in 10 patients.^104^ Interestingly, remission was sustained even after cessation of immunosuppressive therapy in 6 out of 10 responders. In this study, the use of antibiotics seemed associated with failure of FMT. Other, larger trials are underway and the results of these trials are expected soon (NCT03359980). Importantly, transfer of pathogens, including multidrug-resistant bacterial strains^105^ and infectious viruses,^106^ has been described, emphasizing the importance of careful selection and screening of FMT donors.^107^

Finally, not the microbial populations themselves but the metabolites they produce could be replenished. Prebiotics are defined as ‘substrates that are selectively utilized by host microorganisms conferring a health benefit’,^108^ and postbiotics are the bacterial components and metabolites that exert beneficial effects on other microbes, the gut epithelium, and the immune system.^109^ Given the reciprocal effects of pre- and postbiotics on homeostasis and composition of the microbiome, and on health-promoting factors produced by the microbiome, this distinction is somewhat arbitrary however.^84^A few groups have studied the effects of supplementation of microbiome products in allogeneic HCT recipients.^110^ Adequate intake of glutamine, fiber, oligosaccharides and resistant starch mixtures decreased diarrhea, mucositis, and bacteremia, and improved survival.^111–113^ Whether pre- and postbiotic therapy can restore healthy microbial populations, or whether additional support, for example in the form of FMT, is needed remains unanswered.

## Conclusion

Efforts of the last couple of years have revealed important associations between the status of the gut microbiota and the prognosis of allogeneic HCT recipients. Outlines of the dysbiotic fecal microbiome in these patients have been drawn and the first careful steps have been taken to apply this knowledge in a clinical, interventional setting. Moving forward, there are several questions that will need to be answered.

First, a better understanding of the resilience of the microbiome is needed. Recent reports suggest that microbiome damage incurred upon transplantation is not reversible and persists over time.^27,48^ This is in contrast to observations in otherwise healthy individuals, in whom a single insult to the microbiome (e.g. course of antibiotics) induces microbiome damage that is reversible, and warrants further investigation into the causes of the irreversible nature of transplantation-related microbiome damage.^93^ Possible explanations could be persisting adaptive immunodeficiency or the permanent deletion of specific bacterial, archaeal, or viral species or populations that have a detrimental impact on microbiome homeostasis.^45^ With this knowledge, randomized controlled trials can be designed to prevent permanent damage to the microbiome. Additionally, we have to gain more insight into the mechanisms by which intestinal microbiota influence transplant-related complications to design more specific and tailor-made interventions.

Taken together, better care for the microbiome before and after allogeneic HCT, in addition to early interventions to repair microbiome damage after transplantation may significantly reduce transplantation-related morbidity and mortality, and improve allogeneic HCT outcomes in the short and long run.
